# Effect of protein on the thermogenesis performance of natural rubber matrix

**DOI:** 10.1038/s41598-020-73546-7

**Published:** 2020-10-02

**Authors:** Yue-Hua Zhan, Yan-Chan Wei, Jing-jing Tian, Yuan-Yuan Gao, Ming-Chao Luo, Shuangquan Liao

**Affiliations:** Key Laboratory of Tropical Biological Resources of Ministry of Education, School of Materials Science and Engineering, Hainan University, Haikou, China

**Keywords:** Materials science, Polymer chemistry

## Abstract

Under high-speed strain, the thermogenesis performance of natural rubber products is unstable, leading to aging and early failure of the material. The quality of rubber latex and eventually that of the final products depends among others on the protein content. We found that when the protein is almost removed, the heat generated by the vulcanized rubber increases rapidly. After adding soy protein isolate to the secondary purification rubber, the heat generation of the vulcanized rubber is reduced, and the heat generation is the lowest when the added amount is 2.5–3.0 phr, which on account of protein promotes the construction of a vulcanization network and increases the rigidity of the rubber chain, resulting in a decrease in the potential frictional behavior of the rubber chain during the curl up-extension process.

## Introduction

Natural rubber (NR) has been widely used in various fields as a natural bio macromolecular material. Its excellent comprehensive performance is contributed by the unique internal network structure and non-rubber component^1–5^. However, heat generation often occurs during fatigue cycling due to NR’s damping hysteresis effect ^6–8^. The high temperature generated by local heat easily accelerates rubber aging^9,10^, which results in great strength reduction. Even thermal degradation of rubber occurs. Therefore, the thermogenesis performance of products based on NR matrix is an important indicator for evaluating the performance of materials. The lower thermogenesis is, the longer is the service life of the products and the higher is the safety factor.

Since NR is one of the main raw materials of tires, the rubber matrix should have properties such as low thermogenesis and long service life during fatigue cycling. Most reports on thermogenesis performance of rubber products focus on composites with carbon black as the filler^11–14^. The main research direction is the interaction among fillers and the rubber matrix, type of fillers and the dispersion degree of fillers in the rubber matrix to heat generation. In addition, there are some ways to prepare low thermogenesis products by adding high thermal conductive fillers to the rubber matrix^15–18^ and improving the compatibility of the fillers with the rubber matrix. Existing research results show that fillers have an important effect on the thermogenesis performance of composites. However, the contribution of the NR matrix itself to thermogenesis cannot be ignored. As is known to us, NR is a typical viscoelastic material, it has serious hysteresis under high-speed strain, which causes friction between molecular chains and energy loss^19–21^. Especially under harsh service environment, such as aviation tires, tank tracks, etc., it is necessary to use higher quality rubber products. At this time, the material need to meet the service requirements under extreme conditions, simply optimizing the filler performance is not enough. There is also a need to optimize the NR matrix to produce low thermogenesis and high strength rubber products.

The mass proportion of protein in NR is about 1.5–4 m%, which is the most representative of non-rubber components^22^. Our research found that proteins and phospholipids act as sacrificial network in the NR matrix, thus enhance the network structure toughness and strength^23,24^. Moreover, it has always been difficult to study the effect of a single non-rubber component on the rubber properties. Because components of NR are more complex, and it is difficult to accurately purify or remove a component during sample processing. In this paper, in order to remove the effects of the loss of other non-rubber components (acetone soluble, water soluble and inorganic salts etc.) on the thermogenesis performance of NR matrix during centrifugation, we add soy protein^25–27^ to the secondary centrifugal rubber to verify the effect of protein on thermogenesis performance. The structure of soy protein isolate is similar to that of NR endogenous protein. They are both plant proteins, and alpha globulin accounts for about 90%^28,29^. This paper provides a theoretical basis for the preparation and screening of low thermogenesis rubber matrix.

## Results and discussion

### Deproteinization section

#### Effect of protein removal on thermogenesis performance of NR matrix

**Figure 1:**
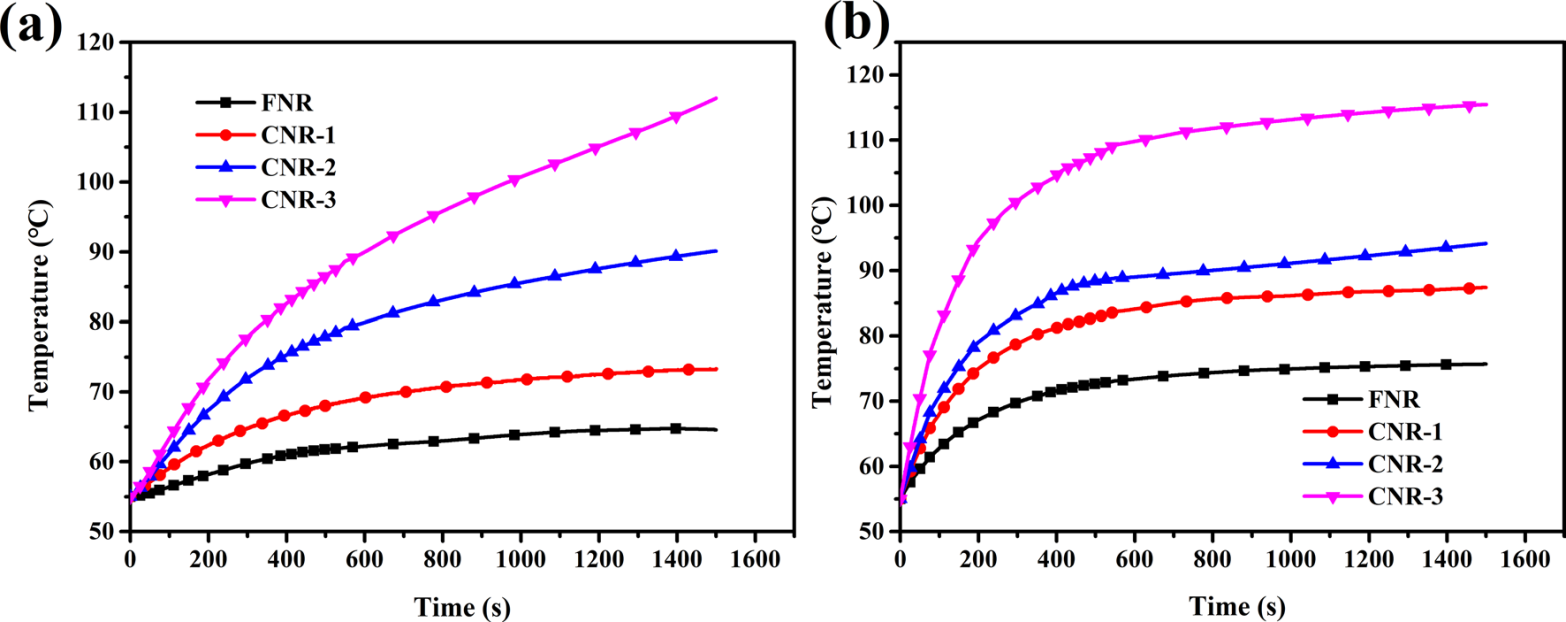
Temperature rise curve of FNR, CNR-1, CNR-2 and CNR-3. (**a**) eXternal of the sample (**b**) internal of the sample.

The temperature rise of the sample is shown in Fig. 1. As the protein content of the NR matrix decreases, the temperature rise of the sample under cyclic stress gradually increased. It can be seen from Fig. 1a that the external temperature rises of FNR, CNR-1, CNR-2, and CNR-3 were 9.6 °C, 18.3 °C, 35.1 °C, and 57.0 °C. Among them, CNR-3 has the highest thermogenesis, which has increased by 47.4 °C compared to FNR, and its temperature still has big uptrend with time. As shows in Fig. 1b, the internal temperature rises of FNR, CNR-1, CNR-2, and CNR-3 were 20.7 °C, 32.4 °C, 39.1 °C, and 60.4 °C. Among them, CNR-3 has the highest thermogenesis, which is 39.7 °C higher than FNR. This is due to the fact that the protein in the natural latex is decomposed into amino acids after enzymatic hydrolysis, which is removed in layers after high-speed centrifugation^30^. The loss of protein will affect the vulcanization kinetics^31,32^, resulting in a weakening of the interaction force between the molecular chains^33^, an increase in the curl up-extension between the molecular chains and in heat dissipation.

#### Effects of protein removal on the composition and structure of NR matrix

**Figure 2:**
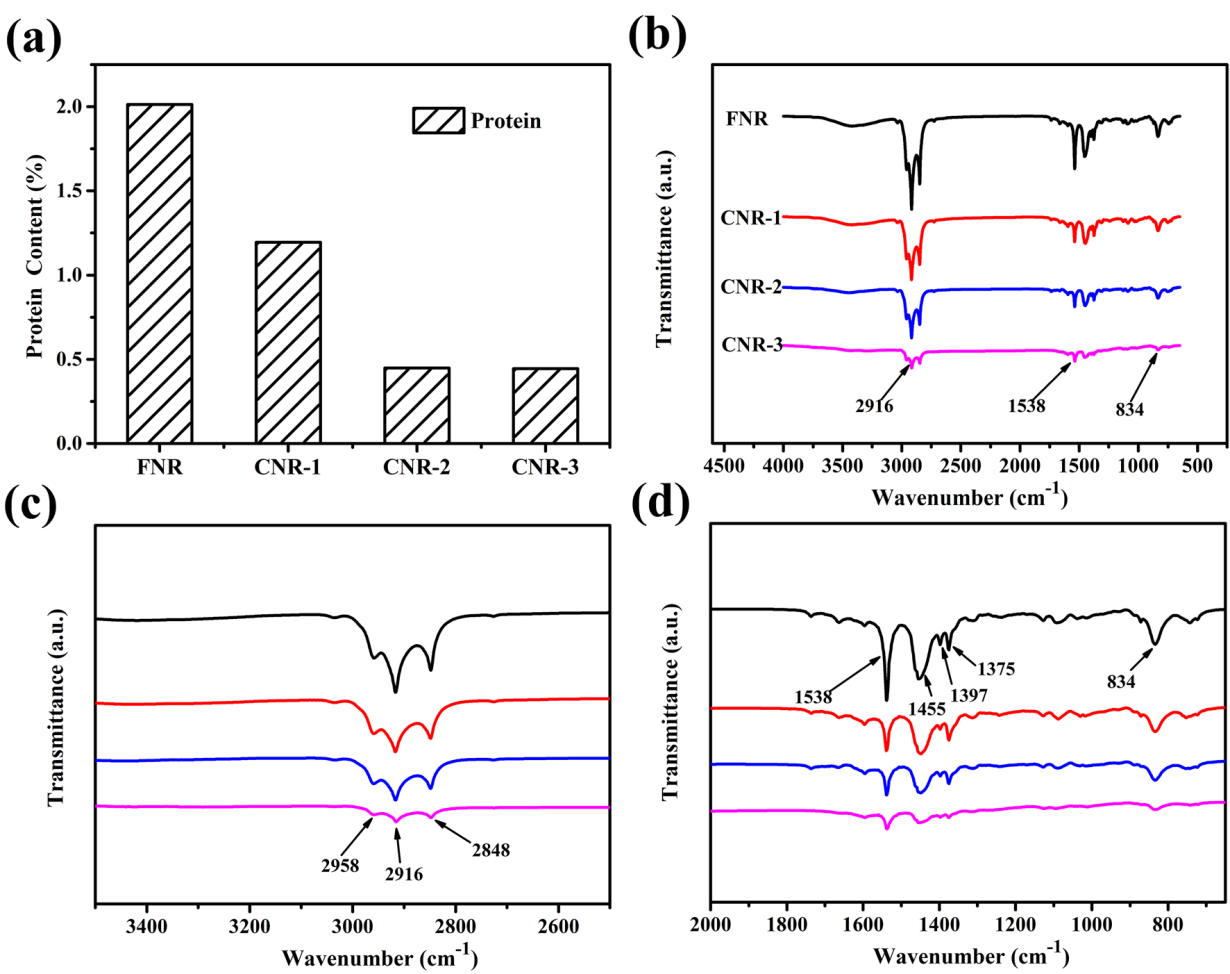
(**a**) Protein content of the sample. (**b**–**d**) FTIR spectra of the sample.

As shown in Fig. 2a, as the number of centrifugations increases, the protein content of the sample decreases rapidly. The protein content of FNR is 2.01 m%, and CNR-3 is reduced to 0.44 m% (weight percentage). The hydrolysis reaction will degrade the bound protein into small molecule amino acids. After centrifugation, the small molecule amino acids and some free water-soluble proteins were separated from the polyisoprene chain. The more centrifugation times, the lower is the protein content in the latex. The infrared spectra of the samples of different contents of protein are shown in Fig. 2b. Because the experimental conditions are consistent, quantitative comparison can be made. The amide I vibration peak at 1630 cm^−1^ is weaker, which is masked by the C=C peak. Therefore, the 1540 cm^−1^ amide II characteristic peak was used to characterize the protein. As shown in Fig. 2d, as the number of centrifugations increases, the intensity of the 1540 cm^−1^ peak weakens significantly. Although enzymatic hydrolysis and centrifugation can effectively remove proteins in natural latex, centrifugal treatment may also cause the loss of other small molar mass non-rubber components (acetone soluble, water soluble and inorganic salts etc.). As the number of centrifugations increases, the intensity of these peaks (marked in the Fig. 2c,d) changed significantly, indicating that it cannot be inferred that only the protein is removed, and it may be accompanied by the loss of other non-rubber components. Therefore, centrifugation leads to an increase in thermogenesis, which cannot be attributed to protein alone.

### Analysis of the effect of protein removal on thermogenesis performance

**Figure 3:**
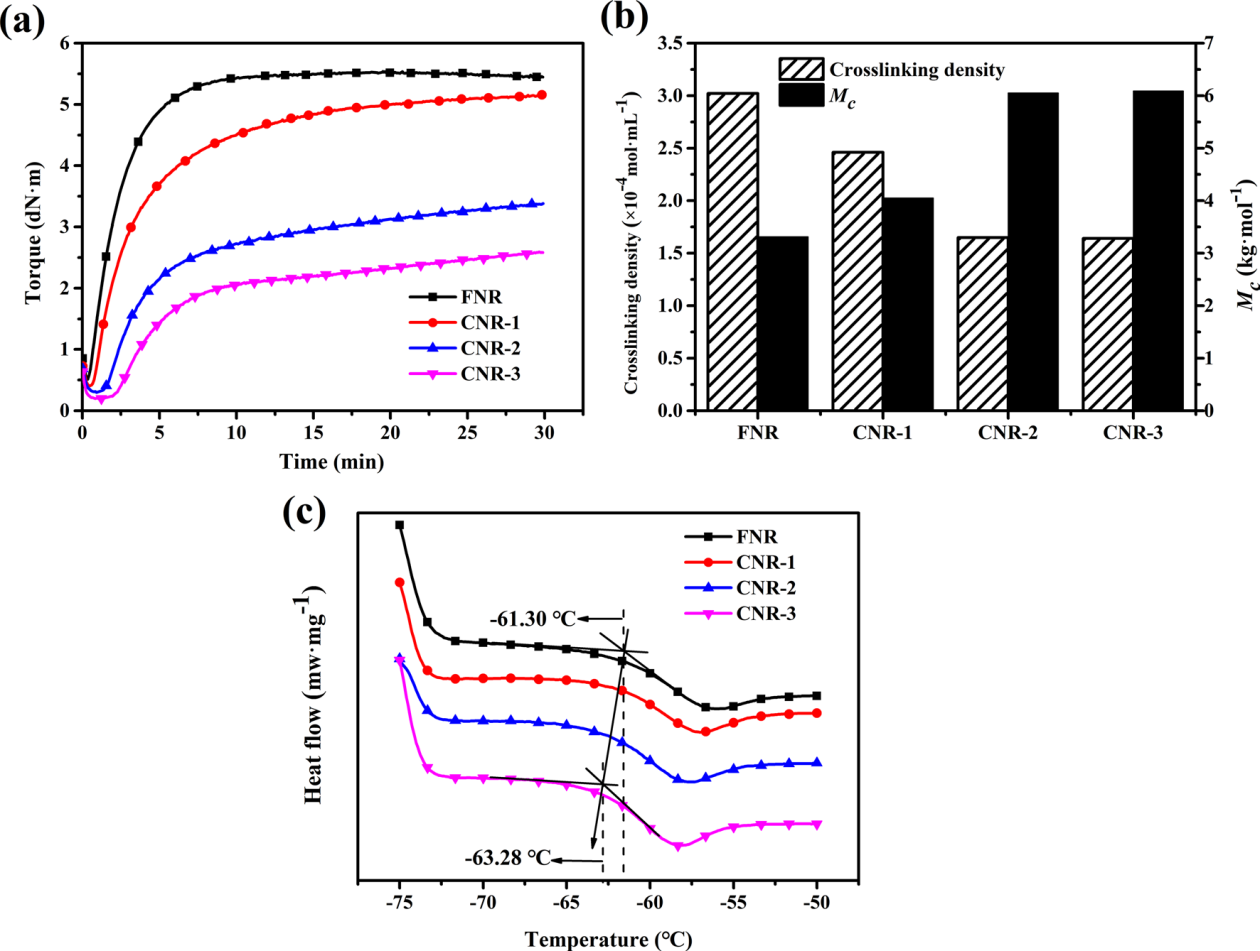
(**a**) Vulcanization curve of the sample. (**b**) Crosslink density and M_c_ of samples. (**c**) Glass transition temperature of samples.

The protein removal has a great effect on the vulcanization kinetics of NR^32^. As shown in Fig. 3a, the maximum torque value (M_HR_) and the positive vulcanization time (t_90_) of the samples significantly changed. The M_HR_ of FNR was 5.44 dN^.^m, and the CNR-3 was reduced to 2.58 dN m. T_90_ of FNR was extended from 5.2 to 20.3 min. It shows that as the protein content decreases, vulcanization kinetics is significantly weakened. The delay of the vulcanization process leads to poor compactness of the chemical cross-linking network, and the mutual binding force between the molecular chains will also be weakened accordingly The cross-linking density of the vulcanized sample was shown in Fig. 3b. The length of the segment between the adjacent crosslinking points increases. As the crosslinking density decreases, the compactness of the vulcanization network decreases and the flexibility increases. As shown in Fig. 3c, the removal of the protein will cause the glass transition temperature of the sample to move at a low temperature (− 61.35 °C to − 63.28 °C), it shows the movement of molecular segment will become easier. It can be inferred that when loaded the same external cyclic stress, the molecular chain of CNR-3 has a lower rigidity and resistance to deformation than the FNR vulcanization network, and the thermogenesis by friction between the chains increases, resulting in more hysteresis energy dissipation and heat generation.

**Figure 4:**
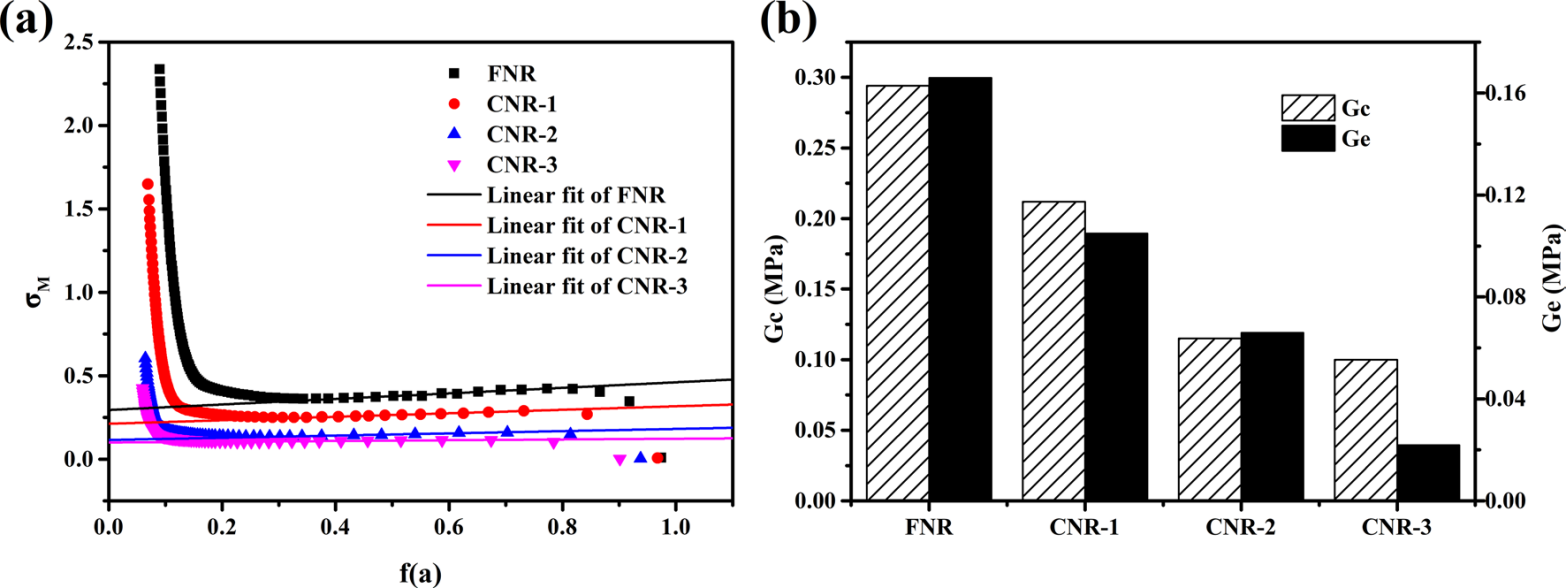
(**a**) Modified stress–strain curves due to the tube model. (**b**) Calculated modulus contributed by entanglement.

Our recent research found that protein constitutes a sacrificial network embedded in the cross-linked network, which breaks preferentially upon deformation to dissipate energy^23,24^. So we can infer that when stress is loading, the preferential break of the protein network will absorb part of the energy to reduce heat generation. Moreover, the protein free in the NR matrix similar to filler will attach to the polyisoprene chain in the form of entanglement-like^22,34,35^. We have been proven that entanglement restricts the movement of molecular chains thus reduces heat generation^7^. To better understand the effect of free protein on the thermogenesis performance of NR matrix, it is necessary to explore the influence of entanglement-like on the thermogenesis in the vulcanization network. In this paper, we use a tube theory^36–41^ applicable to vulcanized rubber to calculate the degree of contribution of topological entanglement in the rubber matrix to the vulcanization network modulus.

1a $${\upsigma }_{M}=\upsigma /(\mathrm{\alpha }-{\mathrm{\alpha }}^{-2})={G}_{c}+{G}_{e}f\left(\mathrm{\alpha }\right)$$

1b $$f\left(\mathrm{\alpha }\right)=\frac{\beta }{2}\frac{{\mathrm{\alpha }}^{\beta /2}-{\mathrm{\alpha }}^{-\beta }}{{\mathrm{\alpha }}^{2}-{\mathrm{\alpha }}^{-1}}$$

where σ_M_ is the reduced stress; σ is the actual stress; G_e_ is the elasticity modulus contributed by the restriction effect of entanglement points; β is an empirical parameter to describe the relationship between a deformed tube in a stretching state and an un-stretched tube in an equilibrium state. In unfilled rubbe, β is assigned 1^37^. α is the ratio of the stretched length to the original length. The value G_e_ is the slope of the linear fit curve derived through fitting points with their X coordinate range being 0.4–0.7 in the Eq. (1a)^42^. The calculation result is shown in Fig. 4, with the protein removal, the modulus contributed by physical entanglements decreases in order, which are 0.166, 0.105, 0.066, 0.022 MPa, respectively. G_c_ also decreased significantly, which represents elastic modulus resulting from the contribution of chemical cross-linking^43^. By combination with Fig. 1, it can be shown that the greater the number of entanglement points, the lower the thermogenesis. it can be attributed to the entanglement-like points of free protein in the NR matrix can "pinned" the molecular chains^7^, limiting the movement of molecular chains, reducing mutual friction between molecular chains and thermogenesis.

**Figure 5:**
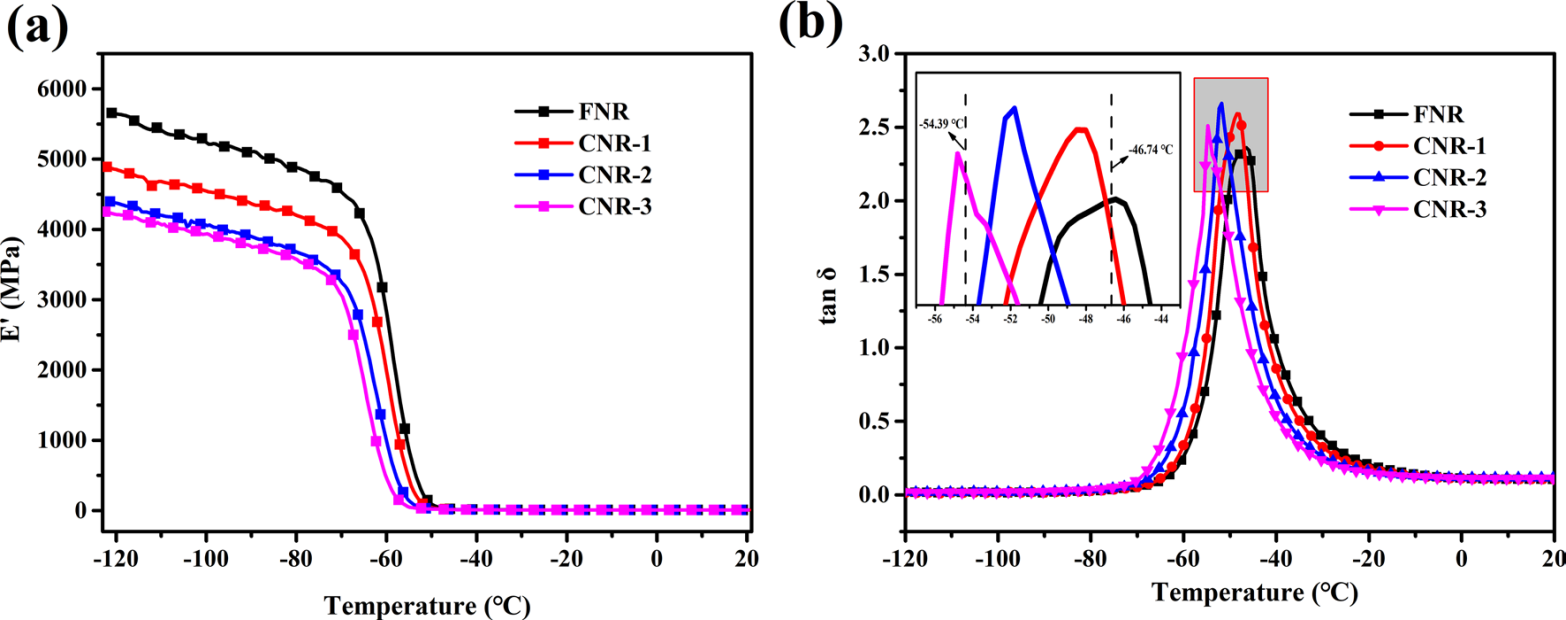
(**a**) The temperature dependent curves of E’. (**b**) The temperature dependent curves of tanδ.

Figure 5 shows the storage modulus (E’) and loss factor (tanδ) of the sample under temperature scanning. Figure 5a shows that the E’ of FNR is the largest. As the protein content decreases, E’ decreases significantly. It shows that the interaction force between the molecular chains gradually decreases, and the strength of the sample network becomes weak. As shown in Fig. 5b, as the protein content decreases, the peak of tanδ moves towards low temperature (− 46.74 °C to − 54.39 °C). This can be inferred that the increased flexibility of the molecular chain is attributed to the decrease in crosslinking and entanglement points, resulting in a decrease in the restriction on the molecular chain. The reason for the inconsistency with Fig. 3c is that the sample tested by DSC is unvulcanized rubber.

**Figure 6:**
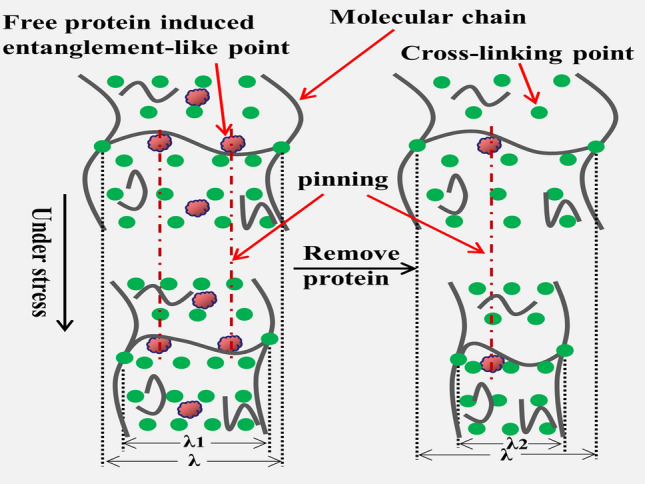
Schematic of vulcanization network of NR matrix with different protein content under the same stress.

The removal of protein causes a significant increase in the thermogenesis of the NR matrix for two reasons:

1. Protein can promote the formation of the vulcanization network, increase the compactness of the cross-linked network. The enhancement of the interaction force between molecular chains makes movement of molecular chains more difficult, and the heat generated by its friction behavior is reduced accordingly.
2. We recently observed the spatial organization of non-rubber components through high-resolution TEM and found that the protein formed a sacrificial network tangled in a cross-linked network^23,24^. According to the calculation of the tube model, it can be known that the free protein can be effect as the entanglement-like point, which can “pinning” the molecular chain and restrict the movement of the molecular chain. As shown in Fig. 6, under the same stress, the NR matrix vulcanization network with less protein content deforms more (|λ − λ_2_| > |λ − λ_1_|). The friction resistance overcome during the molecular chain curl up process became larger, and energy loss increases in the process of overcoming resistance, which increases the heat generated by the rubber matrix.

### External protein validation section

In addition to protein, the NR matrix also contains other non-rubber components such as acetone soluble, water soluble and inorganic salts etc.. The centrifugal treatment of natural latex will cause the loss of other non-rubber components in rubber, and it cannot be accurately inferred whether it is the effect of protein alone. In order to more accurately investigate the effect of protein on the thermogenesis performance of NR, gradient protein was added to CNR-2 during the mixing process for verification. It can be seen from Fig. 2a that the protein removal of the sample is relatively complete. If the thermogenesis of the sample decreases with the increase of the content of protein added, the effect of protein on the thermogenesis performance of NR can be demonstrated.

#### Effects of external protein on the composition and structure of NR

**Figure 7:**
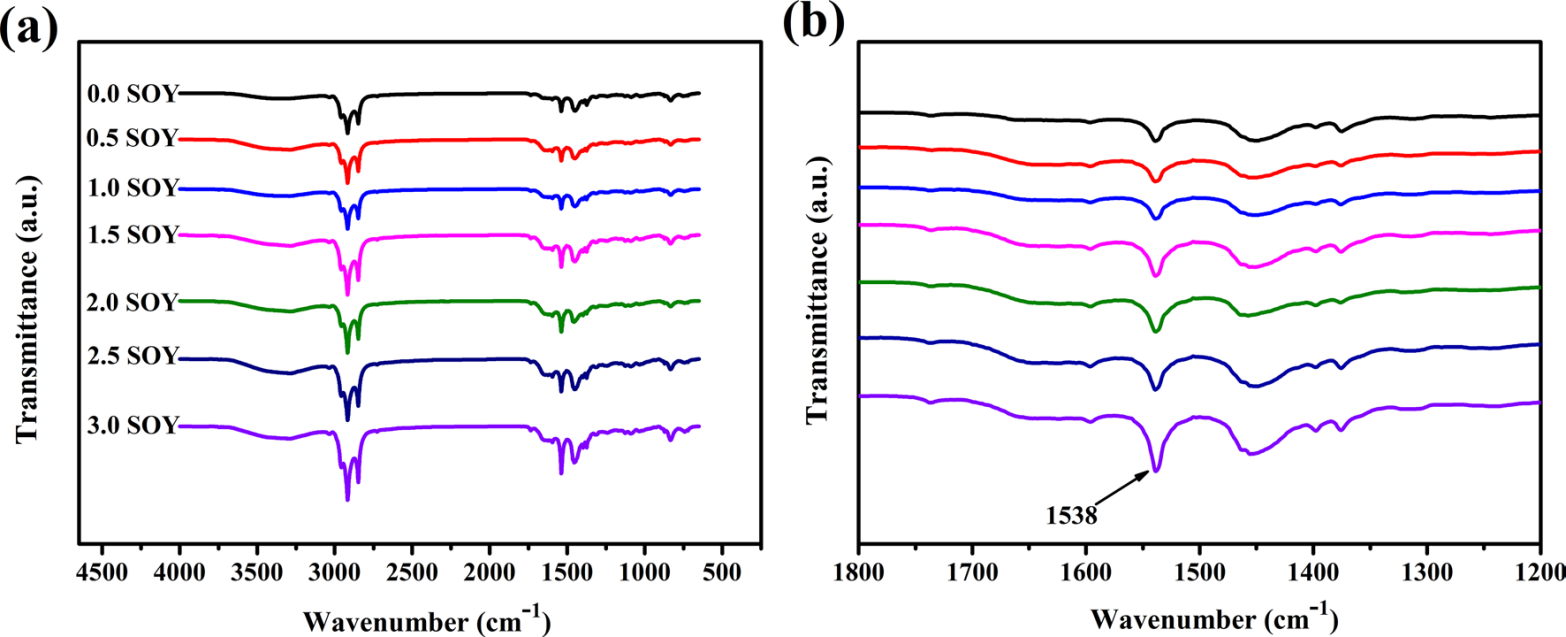
FTIR spectra of external protein samples.

Figure 7a is the infrared spectrum of the sulfurized sample after adding soy protein isolate. As shown in Fig. 7b, as the external protein content increases, the characteristic peak of 1540 cm^−1^ derived from amide II is enhanced. Indicating that with the mixing and vulcanization processing, soy protein was compatible with NR matrix.

#### Effect of external protein on thermal performance of NR matrix

**Figure 8:**
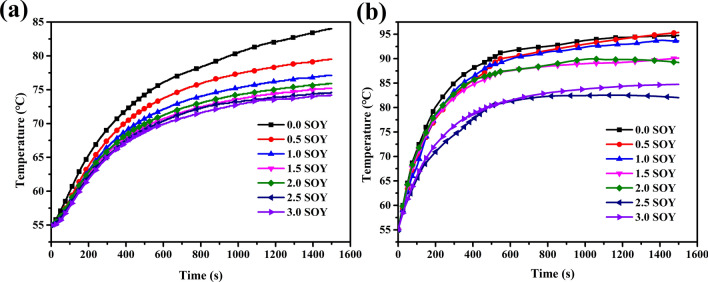
Temperature rise curve of external protein samples. (**a**) External of the sample, (**b**) internal of the sample.

As the amount of protein added increased, the temperature rise of the sample was clearly decreased. As shown in Fig. 8a, The external temperature rises of the samples xSOY (x represents the mass fraction of soy protein added, and the numerical arrangement increases in order) were 29 °C, 24.5 °C, 22.1 °C, 20.2 °C, 20.9 °C, 19.6 °C, and 19.2 °C, respectively. Among them, 3.0 SOY has the lowest external temperature rise, which is 9.8 °C lower than 0.0 SOY. It shows that the reduced amplitude of heat generation tends to be gentle as the amount of added protein increases. As shown in Fig. 8b, as the amount of protein added increases, the internal temperature rise of the sample is 39.7 °C, 40.4 °C, 38.6 °C, 36.1 °C, 34.2 °C, 27 °C, 29.7 °C. Among them, 2.5 SOY has the lowest internal temperature rise, which is 12.7 °C lower than 0.0 SOY. It can be inferred that with the addition of protein, the thermogenesis of rubber can be suppressed, but it is not better to add as much as possible. It can be seen from Fig. 8b that the internal temperature rise of 3.0SOY (29.7 °C) is higher than that of 2.5SOY (27 °C). This attributed to when a large amount of protein is added, it will lead to poor compatibility between the protein and the rubber matrix^22^.

## Materials and methods

### Materials

Fresh NR latex (Total solid content: 43.3%, dry rubber content: 41.4%, ammonia content: 0.23%) was provided by China Hainan Rubber Industry Group Co., Ltd.; (China); Zinc oxide (99%), sulfur (99%), stearic acid (98%) and 2-Mercaptobenzothiazole (98%) were provided from Shanghai Yuanye Biotechnology Co., Ltd. sodium dodecyl sulfate (SDS) (99%), Alkaline protease (≥ 500 units/mL) and isolated soy protein (90%) were provided from Macklin Reagent Co.

### Preparation of vulcanized samples

Firstly, we measured the dry rubber content of fresh latex and diluted the fresh NR latex with distilled water to 25 m% dry rubber content, then add 0.5 m% SDS and 0.04 m% alkaline protease, mixing and stirring at 37 °C water bath for 24 h. Secondly, centrifuge it at 12,000 r/min at 4 °C for 1 h, and remove the upper emulsion, and repeat the above-mentioned centrifugation treatment 1 to 3 times to obtain raw rubber with different protein content. Third, dry rubber was obtained by natural film coagulation at room temperature. Finally, the compounds is obtained by mixing curating agents and row rubber on mixing rolls, then subjecting to compression in cylindrical cavity at 145 °C for double t_90_. Then primary centrifugal vulcanized rubber (CNR-1), secondary centrifugal vulcanized rubber (CNR-2), tertiary centrifugal vulcanized rubber (CNR-3) was obtained, and vulcanized rubber that have not been centrifuged are labeled FNR. Add 0.0, 0.5, 1.0, 1.5, 2.0, 2.5, and 3.0 phr of soy protein to CNR-2 in the process of mixing, and vulcanized rubber was obtained after vulcanization, which was recorded as 0.0 SOY, 0.5 SOY, 1.0 SOY, 1.5 SOY, 2.0 SOY, 2.5 SOY, 3.0 SOY. The above samples are all taken from the same batch of latex.

Vulcanization formula: NR, 100 phr; stearic acid, 0.5 phr; 2-Mercaptobenzothiazole, 0.5 phr; zinc oxide, 6.0 phr; sulfur, 3.5 phr; total, 110.5 phr.

### Measurements and characterization

**Figure 9:**
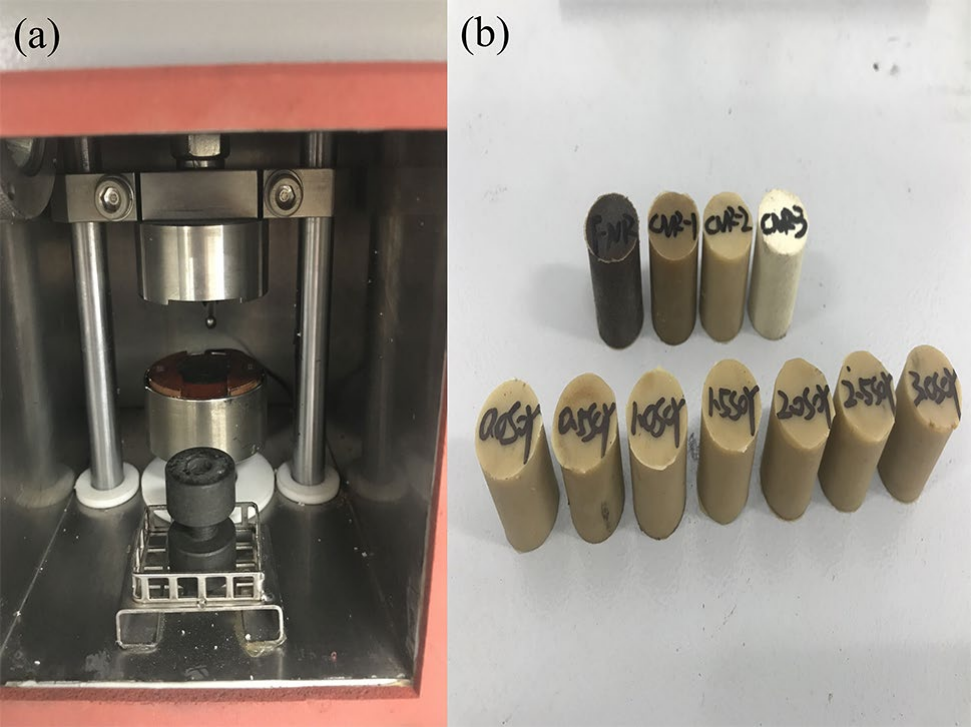
Physical map of (**a**) Instrument cavity and (**b**) vulcanized rubber.

Heat generating test: RH-2000 N thermogenesis analyzer produced by Taiwan Gotech co. was adopt to test the temperature rise of the external and internal part of the sample. Figure 9 is the morphology of thermogenesis test cavity and samples. As shown in Fig. 9a, the temperature inductor at the bottom of the instrument can monitor external temperature changes of sample in a real-time manner. The temperature rise of the internal part of the sample will be monitored by a thermocouple. Test 3–5 times for each parallel sample, and take the curve with the best reproducibility as the result. The sample was compression curing in a cylindrical mold cavity, its specification was shown in Fig. 9b: diameter: 17.8 ± 0.15 mm; height: 25 ± 0.25 mm; hardness: lower than 85 IRHD; constant temperature of the test die cavity: 55 °C; frequency: 30 Hz; prestress: 1 MPa; stroke: 4.445 mm.

FTIR spectra analysis: TENSOR27 FTIR spectrometer produced by German Bruker co. was used to analyze the composition and structure of vulcanized rubber. The sample was cut into a thin film, and attenuated total reflection was used to obtain the spectra. The wavenumber range was 4000–650 cm^−1^ and scan 32 times.

Protein content: The K9860 automatic Kjeldahl meter produced by China Haineng co. was used to determine the protein content of raw rubber. The formula for calculating protein content is as follows: ${W}_{\mathrm{Protein}}={W}_{\mathrm{Nitrogen}}\times 6.25$.

Vulcanization curve: The QLB-D type vulcanizer produced by China Jiangdu co. was used to test the vulcanization characteristics of the rubber, and the test temperature is 145 °C. Test 3–5 times for each parallel sample, take the curve with the best reproducibility as the result.

Stress–strain test (used for tube theoretical fitting): The AI-3000 tensile tester produced by the Taiwan Gotech Company was used to determine the tensile properties of the vulcanized rubber. The sample was a dumbbell shaped strip with central dimensions of 25 mm × 6 mm × 1 mm, and the tensile rate was 500 mm/min. Test 3–5 times for each parallel sample, take the curve with the best reproducibility as the fitting data.

Determination of crosslink density: The crosslink density of vulcanized rubber was tested by using German IIC-XLDS-15HT type nuclear magnetic resonance crosslink density. The sample was a thin rectangular slice shape. The test temperature was 80 °C, the frequency was 15 MHz, and the magnetic induction intensity was 315 A/m. Test 5 times for each parallel sample, take the average as the result.

Glass transition temperature: the DSC822/400 differential scanning calorimeter produced by Switzerland Mettler-Toledo was used to test the glass transition temperature of the unvulcanized rubber. The mass of the vulcanized sample was in the range of 6–8 mg, and then put in aluminum positioning crucible. Temperature was increased from − 75 to 25 °C at a heating rate of 5 °C/min in N_2_ atmosphere.

Dynamic mechanical properties: The DMA242C/1/G dynamic thermomechanical performance analyzer produced by the German Netzsch co. was used to test the storage modulus (E′) and loss angle (tanδ) of the vulcanized sample. Temperature rise from − 120 to 20 °C at 5 °C/min heating rate, and frequency is 1 Hz. The sample was a rectangular slice with dimensions of 6 mm × 2 mm × 1 mm. Test 3–5 times for each parallel sample, and take the curve with the best reproducibility as the result.

## Conclusions

There are two factors that influence the heat generation of NR by protein. Firstly, protein tuning network structure of NR from vulcanization kinetics. It promotes the construction of cross-linked networks, reduce the flexibility of molecular chains, and limit the behavior of friction between molecular chains. Secondly, combined with the analysis of the tube model, it is shown that with the removal of the protein, the number of entanglement-like points in the rubber network decreases significantly, resulting in a weakening of the “pinning” effect on the movement of the molecular chain, increasing the heat generation.

## Supplementary information

Supplementary information.
