# Supplementary material for: Effect of protein on the thermogenesis performance of natural rubber matrix
Source: Sci Rep. 2020 Oct 2;10:16417. doi: 10.1038/s41598-020-73546-7 (PMC7532221; doi:10.1038/s41598-020-73546-7)
Supplement: Supplementary file 1 — Supplementary information. [file 41598_2020_73546_MOESM1_ESM.docx]

# Supplementary material

**Effect of protein on the****thermogenesis performance of natural rubber matrix**

**Yue-Hua Zhan^a^, Yan-Chan Wei^a^, Jing-jing** **Tian^a^, Yuan-Yuan Gao^a^, Ming-Chao Luo^a^, Shuangquan Liao*^a^**

^a^ Key Laboratory of Tropical Biological Resources of Ministry of Education, School of Materials Science and Engineering, Hainan University, Haikou, China

^*^Corresponding author: Shuangquan Liao E-mail addresses: lsqhnu@hainanu.edu.cn

In order to verify whether the influence rule of the soy protein on the thermogenesis performance of the rubber matrix is suitable for endogenous protein, NR with different content of endogenous protein was tested for heat generation, and test 3-5 times for each parallel sample, and take the curve with the best reproducibility as the result. The content of endogenous protein was 2.84 %, 2.21 % and 3.77 %, and recorded as 2.84 PRO, 2.21 PRO and 3.77 PRO, respectively. Figure **S1** shows the heat generation curves. As shown in figure **S1a**, after loaded with high-speed cyclic stress, the external temperature rise of 2.84 PRO is the lowest, only 6.7 ^o^C, 2.21 PRO, 3.77 PRO are 7.2 ^o^C and 8.8 ^o^C, respectively. The temperature rise during the test is shown in figure **S1b**. The internal temperature rise of 2.84 PRO is the lowest, only 18.2 ^o^C, 2.21 PRO and 3.77 PRO are 20.3 ^o^C and 19.2 ^o^C, respectively.





**Figure S1**

**

**

**Figure S2**

**

**

**Figure S3**

**Figure caption**

Figure S1. Temperature rise curve of samples with different endogenous protein content. (a) external of the sample (b) internal of the sample

Figure S2. Protein content of xSOY (x represents the mass fraction of soy protein added).

Figure S3. The infrared spectrum of soybean protein isolate
